# Working within the Design Space: Do Our Static Process Characterization Methods Suffice?

**DOI:** 10.3390/pharmaceutics12060562

**Published:** 2020-06-17

**Authors:** Moritz von Stosch, René Schenkendorf, Geoffroy Geldhof, Christos Varsakelis, Marco Mariti, Sandrine Dessoy, Annick Vandercammen, Alexander Pysik, Matthew Sanders

**Affiliations:** 1 GSK, B-1330 Rixensart, Belgium; movosto@gmx.net (M.v.S.); GEOFFROY.Q.GELDHOF@GSK.COM (G.G.); CHRISTOS.X.VARSAKELIS@GSK.COM (C.V.); marco.x.mariti@gsk.com (M.M.); SANDRINE.DESSOY@GSK.COM (S.D.); ANNICK.VANDERCAMMEN@GSK.COM (A.V.); alexander.x.pysik@gsk.com (A.P.); MATTHEW.2.SANDERS@GSK.COM (M.S.); 2 Institute of Energy and Process Systems Engineering, TU Braunschweig, 38106 Braunschweig, Germany; 3 Center of Pharmaceutical Engineering, TU Braunschweig, 38106 Braunschweig, Germany

**Keywords:** process analytical technology, quality by design, dynamic design space, reachability, flexibility, dynamic modeling, critical process parameters, critical quality attribute

## Abstract

The Process Analytical Technology initiative and Quality by Design paradigm have led to changes in the guidelines and views of how to develop drug manufacturing processes. On this occasion the concept of the design space, which describes the impact of process parameters and material attributes on the attributes of the product, was introduced in the ICH Q8 guideline. The way the design space is defined and can be presented for regulatory approval seems to be left to the applicants, among who at least a consensus on how to characterize the design space seems to have evolved. The large majority of design spaces described in publications seem to follow a “static” statistical experimentation and modeling approach. Given that temporal deviations in the process parameters (i.e., moving within the design space) are of a dynamic nature, static approaches might not suffice for the consideration of the implications of variations in the values of the process parameters. In this paper, different forms of design space representations are discussed and the current consensus is challenged, which in turn, establishes the need for a dynamic representation and characterization of the design space. Subsequently, selected approaches for a dynamic representation, characterization and validation which are proposed in the literature are discussed, also showcasing the opportunity to integrate the activities of process characterization, process monitoring and process control strategy development.

## 1. Introduction

The Process Analytical Technology (PAT) initiative, proposed by the FDA in 2004, and the Quality by Design (QbD) concept, which is at the heart of the PAT implementation, are currently changing the ways drugs are manufactured and how manufacturing processes are approved. A particular concept which arose from these activities is that of the process design space. The design space is a multidimensional space, spanned by selected process parameters and material attributes. Operation within the design space yields selected quality attributes of the drug to stay within acceptable ranges (the approach for the selection of process parameters and quality attributes is sketched below). Several publications describe the development of design spaces in the pharmaceutical QbD context [1,2,3,4,5] and a review of the design space development was performed by Debevec et al. However, most of these design space developments use a rather static characterization of the design space, via Design of Experiments (DoEs) and statistical modeling approaches.

Two questions arise when contrasting the operation and control of the process, which are dynamic activities (i.e., dynamic changes in the process parameters within the design space), against the typical static characterization. The first question is: does the employment of a static characterization and representation over-restrict the space that could be used for process control? This is more so, given that dynamic design space can probably result in larger spaces and thus increase efficiency (process capability) (Note that we are assuming that the process is robust, i.e., temporal deviations within the current “standard-static” design space will not necessarily result in significant deviations in the Critical Quality Attributes (CQAs) even though limited understanding of the impact of the process dynamics on the CQAs is available. Cases can be constructed (both theoretical and experimental) to show that temporal deviations lead to a complete change of the process outcome and thus in the CQAs. However, in these cases, the processes are typically operated close to a point at which the process behavior changes significantly, which would typically result in a significant process to process variation and thus would not qualify as a robust process). The second question is: what methods are available or needed to develop dynamic design spaces, such that these spaces can directly be adopted for process control/operation during manufacturing, i.e., merge the developments of the process characterization and process control strategy?

Relatively few publications describe dynamic design space concepts or the development of dynamic design spaces. In what follows, we address the above raised questions in light of the published dynamic design space work.

## 2. Design Space

The ICH guidance Q8 [6] defines the design space as “the multidimensional combination and interaction of input variables and process parameters that have been demonstrated to provide assurance of quality.” Further, it is stated that: “Working within the design space is not considered as a change. Movement out of the design space is considered to be a change and would normally initiate a regulatory post-approval-change process.”

### 2.1. The Current View on the Development and Representation of the Design Space

The exact representation of the design space and what the design space exactly describes seems to be left to the applicant, i.e., “Design space is proposed by the applicant and is subject to regulatory assessment and approval” [6]. However, a common understanding seems to be reached on how the design space should be developed, referred to as the QbD roadmap [1,2,3,4,5,7]. Based on the Quality Target Product Profile (QTPP), the Critical Quality Attributes (CQAs) of the product either at the output of the entire process or after each process unit are identified based on risk assessment approaches. These CQAs can then be used along with prior process knowledge in risk assessment to identify those process parameters and material attributes that are expected to be critical for achieving the desired CQA acceptance criteria, i.e., the critical process parameters (CPPs) and the critical material attributes (CMA). Different levels of the CPPs and CMAs are then usually systematically studied using DoEs. The experimental responses in CQAs observed in the different experiments are subsequently assessed by using analytical approaches, such as response surface models and Analysis of Variance (ANOVA). These approaches yield a process model in which the impact of the CPPs and CMAs on the CQAs is described by a set of algebraic and possibly nonlinear equations, i.e.,

(1) $$\mathrm{CQAs}=f\left(\mathrm{CPPs},\mathrm{CMAs},w\right)$$

where *w* are the process model parameter estimates. The form of this equation seems to be used to describe the design space when filing the design space [2,6,7]. The equation can either describe each unit operation or the entire process [6]. For regulatory approval, the CQAs have to remain within strict limits (that are largely determined from the QTPP obtained through clinical trials or preclinical studies [1] and might, in addition, give margin to analytical variability but are not subject to discussion here). The limits of the CQAs, in turn, impose limits on the CPPs and CMAs, i.e., the set of all values of CPPs and CMAs for which the value of CQAs, calculated with Equation (1), falls within the quality limits are feasible for CPP and CMA combinations. The process design space, i.e., the space in which a certain product quality can be achieved, is therefore described by the process model, Equation (1), together with a set of constraints that specify the boundaries of the CQAs space. How the limits/boundaries of the design space could be identified is described below.

### 2.2. Alternative Representations of Multidimensional Spaces

**Figure 1:**
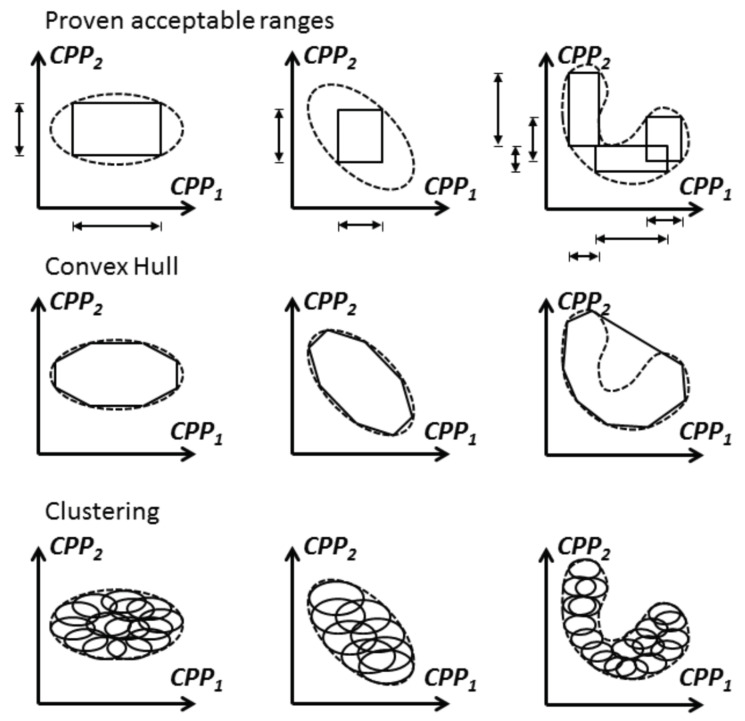
Three stand-alone ways of representing the design space. Dashed line: Explored design space; Continuous line: Design space boundaries described by the chosen representation approach.

For manufacturing purposes, it might be useful to adopt an alternative way for the representation of the design space, e.g., by facilitating operators to check whether the process is progressing within the specified multidimensional space. Specifying ranges for a set of variables defines a multidimensional space (The design space can be represented by proven acceptable ranges, but proven acceptable ranges do not automatically constitute a design space). In two dimensions, this space is a rectangle, in three dimensions a rectangular cuboid and in *N* dimensions (N≥3) a *N*-orthotope. Note that the variables are assumed to be independent when representing the design space as a rectangle, i.e., the dependencies of CPPs or CMAs are ignored. This form of defining a multidimensional space is practical as it is straightforward to check whether a variable is within the space, i.e., whether it is within the defined range or not. It is, moreover, easy to operate within the space as each variable can be moved and thus considered independently (though interaction effects might come into play). In addition, the defined space is convex, i.e., every two points within the space can be connected with a line segment whose points never leave the region. Convexity is an attractive property (though not formally required) since the defined space will not be left when following a linear path from one-set point to another for process control purposes. However, the definition of the range for each variable can limit the space that can be used for control dramatically, as illustrated in Figure 1. Thus, the definition of ranges for each variable is not the most advantageous way to specify multivariate spaces.

A potential alternative is provided by the convex hull concept. A convex hull, as the name already indicates, is a convex multivariate space that is computed from points that can be found within the explored space, e.g., experimental points. More specifically, given a set of points, the convex hull is defined as the smallest convex set that contains all of these points. These points could, for instance, stem from experiments or simulation studies. It has been shown by Kahrs and Marquardt [8] that a convex hull which is expressed as an intersection of half-planes (e.g., using the quickhull algorithm for convex hulls [9]) can be reformulated into a set of linear inequality constraints

(2) Ax≤b,

where *x* is the current operating point (a vector), *A* is a matrix that defines the position of *x* within the space, and *b* are the boundaries of the space. This means it can easily be checked whether or not a new point falls within the convex space (i.e., if the point *x* falls within the defined space then Ax≤b else Ax is greater than *b*).

However, if the points are unevenly spread in the space or if the space of interest is non-convex (as in the A-Mab case [10]) then the convex hull might not be well suited for the representation of the explored region (the space could be broken down into a subspace to obtain a convex space); see Figure 1 for illustration. In such cases, clustering approaches could be more suitable to characterize the explored space (Please note that clustering is an NP-hard problem. Although empirical algorithms do exist, the problem becomes computationally challenging as the dimensions increase). Once clusters have been defined, new points can be assigned to their corresponding cluster by computing their Euclidean distance to all clusters; numerically, this is a straightforward operation. However, the clustered space is not necessarily convex, even when looking at convexity per cluster, and it also depends on the definition of a set of parameters, e.g., number of clusters or cluster width. In addition, some distance from the clustered space will have to be defined that determines the boundaries of the design space, which might not be trivial.

Another approach proposed by Castagnoli et al. [11] is to define a confidence level on the CQAs (e.g., 90% chance of meeting all CQAs) and to use the prediction of the model developed for linking the CPPs and CQAs to check whether with the current CPPs the CQAs are met. This approach, also referred to as the parametric control, is to a large extent ad hoc and as such subject of debate as it requires the definition of the confidence levels, which might also not be trivial.

### 2.3. Operation within the Design Space

Moving within the design space is not considered a change in the process and, in principle, does not require post-approval. Real-time monitoring and control strategies need to ensure that deviations in CPPs/CMAs (and/or CQAs, if possible to monitor them directly in real-time) are observed and can be acted on, bringing the process back to “normal/optimal” operation. The criticality of process parameters and material attributes, which was assessed during the risk assessment, as well as the impact of the CPPs and CMAs, identified during the DoE-data analysis studies, should be considered for the development of the control strategy. It is eminent that parameters and attributes that are critical and have more impact should be controlled precisely and timely [4]. Interactions between the process parameters/material attributes are possible, and the control strategy must also account for those. Both the weighting of the parameters/attributes and the integration of interactions can be achieved by advanced process control approaches [12,13], such as Model Predictive Control (MPC) [14,15], which have been widely and successfully adopted in other industries [16].

Based on the comments above, reliable methods of uncertainty analysis and process control are indispensable for a reliable identification of the dynamic design space and its practical realization, respectively. Thus, the current state-of-the-art, as well as the need for research, of these methods deserve a more detailed discussion.

#### 2.3.1. Uncertainty and Sensitivity Analysis

External disturbances (e.g., raw material variability or environmental factors) and process uncertainties could affect the product quality, which could then lead to the rejection of the manufactured pharmaceuticals and operational failures [17,18]. The reliability of the designed processes under different conditions and disturbances is called robustness. Robustness, in turn, can be achieved both by advanced control concepts as well as by solving process design optimization problems under uncertainties [19,20]. Optimization problems that consider process performance and robustness are the starting point of the dynamic design space approach to finding solutions for real plants of industrial relevance while minimizes the risk of producing off-spec pharmaceutical products. Furthermore, the uncertainties should also be taken into account in the controller synthesis.

For instance, robust optimization concepts have been widely used to design upstream synthesis units [21,22,23] and downstream separation units [17,21,23,24] for pharmaceutical manufacturing processes. Here, worst-case and the possibility-based approaches are a good choice for coarse uncertainty expressions, but could lead to conservative results [25]. Alternatively, probability-based concepts that provide detailed parameter uncertainty information regarding probability density functions have attracted considerable attention over the last decade; see [26] and references therein.

To screen out the CMAs and CPPs that have substantial impacts on the final drug product quality can be systematically identified via sensitivity analyses (SA) [19,27]. With the SA, not only can the identified, relevant variables serve for a better process understanding or process design, they can also be helpful for the correct control loop synthesis and parameterization. Methodologically, the global sensitivity measures, in particular, have proven to be particularly meaningful in the area of highly complex manufacturing processes for pharmaceuticals [28]. For example, fluctuations in the starting material and their effects on the upstream and downstream processes up to the final product quality can be comprehensively recorded, understood, and, if necessary, compensated with suitable countermeasures [19,27,29]. For efficient utilization of the uncertainty and sensitivity analysis for the dynamic design space, however, the underlying algorithms must be further developed.

On the one hand, a better basic understanding of the methods themselves as well as user-friendly tools that help to implement and reproduce the results are needed (Note that in practice, SA has also been incorrectly implemented and misinterpreted in the recent past due to the lack of these tools in the various disciplines, especially in the field of process engineering).

On the other hand, the computing times required for the uncertainty analysis and SA must be further reduced. Besides advanced sampling methods, especially methods based on easy to evaluate surrogate models (e.g., neural networks and polynomial functions) seem promising [26]. Likewise, the exact description of the uncertainties themselves must become more important in the focus of the dynamic design space analyses. Especially in the pharmaceutical sector, for instance, the problem of batch-to-batch variability is well known. These uncertainty effects, as well as their interactions, must be adequately integrated into future studies as discussed, for instance, in [22,26].

#### 2.3.2. Control and Systems Theory

From the previous presentation on dynamic design space, it became clear that both control and systems theory play an important role. The concept of dynamic design space can only be practically implemented with advanced control concepts; see [30,31,32] and references therein. Assuming functioning control loop structures for the individual process units, it is precisely the higher-level control architecture that enables flexible pharmaceutical manufacturing processes that adapt to disturbances while complying with the required final CQA acceptance limits [33,34,35,36,37].

In addition to real-time capable implementation, distributed control concepts are required to guarantee stability. Model-based control concepts, as well as reinforcement control, are particularly suitable for this purpose and are already being used in a wide range of application areas—including safety-critical processes [38,39,40]. However, there are only a few documented examples of this in the pharmaceutical sector [36,41]. For instance, in Kager et al. [42], the authors discuss based on validation experiments the added value of MPC compared to the classical feedback control approach for a (bio)pharmaceutical case study. Most scenarios are limited to individual process steps and do not specify or prove strict stability criteria for given controller implementations. Reinforcement control algorithms with guaranteed stability conditions and MPC concepts under uncertainties must, therefore, be transferred and validated to the requirements in the field of pharmaceutical process engineering [32,43,44]. The system properties also play an essential role in the success of process control and monitoring in the context of dynamic design space.

It is therefore desirable that the aspects of controllability and observability are already considered in the process design phase [45,46]. For this purpose, the corresponding systems theory concepts must, on the one hand, be made applicable to the complexity of the nonlinear process models of pharmaceutical process engineering, and suitable metrics must be defined for integration into the model-based process design [47,48]. In the literature, current work is already pointing in the right direction, but the influence of process nonlinearities and process uncertainties has not yet been fully taken into account; see [48] and references therein. Other studies already link these systems theory aspects with model-based process design concepts [17,49]. Despite the promising reported results, the analyses are limited to simple control and process monitoring tasks. For industry-related applications and the description of holistic process chains, the methods of process monitoring and control must be combined with methods of model reduction. Multivariate statistical models (e.g., principal component analysis (PCA) or partial least square (PLS)) can reduce the dimension of complex CMA, CPP, and CQA interaction models by exploiting correlation and dependencies measures [33,50,51,52]. Similar to uncertainty analysis, see Section 2.3.1, CPU-friendly surrogate models also play an essential role when it comes to real-time algorithms [53,54,55] for the implementation of model-based dynamic design space strategies. For successful exploitation of the dynamic design space, these concepts have to be further developed and adapted to higher-level control structure, process monitoring concepts, and multi-unit design space problems.

In summary, these mentioned concepts require dynamic process models that describe the impact of the attributes/parameters on the process dynamics and control actions—the static model used for the design space, Equation (1) cannot be used for this purpose. In light of the expressions such as “moving within the design space” that indicate a dynamic evolvement, a dynamic modeling approach also seems to fit within the thinking frame of the proponents of the design space concept.

## 3. A Dynamic Design Space Model

Dynamic process models have been extensively studied in the field of advanced process control. A general way to present such models is via a system of nonlinear Ordinary Differential Equations (ODEs)

(3) $$\frac{dx}{dt}=g\left(x,\mathrm{CPPs}(t),\mathrm{CMAs}(t),p\right)$$

where *x* represents the process states and *p* a set of parameters. Note that ODEs are typically based on mechanistic (first-principles) models, i.e., models that reflect the underlying physicochemical mechanisms [27,54]. However, while relevant process steps are often still not fully understood, data-driven models also play an important role here. Especially hybrid models [54,56], i.e., the combination of mechanistic and data-driven models, might be crucial for the implementation of dynamic design space strategies. The CPPs and CMAs can be interpreted as a set of control inputs that are also time-dependent. The time trajectory describes the evolution of the process state in time, which might be dependent on the process states itself. The CQAs can then either be modeled as process states or they can be modeled separately using an additional equation, such as:

(4) $$\mathrm{CQAs}\left(t\right)=h\left(x(t),\mathrm{CPPs}(t),\mathrm{CMAs}(t),\omega \right)$$

with ω a set of parameters that might be additionally needed. Note that Equation (4) takes dependencies of the CQAs on the process states x(t) explicitly into account. In both cases (CQAs being modeled either via Equation (1) or Equation (4)), the CQAs can depend on the process state. This dependence is indirectly considered when deriving models of the form of Equation (1), but for this, it is essential that the initial process states are similar at the beginning of the process and further that throughout each state of the experiment, the CPPs and CMAs are constant. Neglecting this dependence is generally not critical. However, one can think of cases where it becomes substantially more relevant (e.g., variations of the raw materials), and this would have to be picked up by the monitoring strategy.

**Figure 2:**
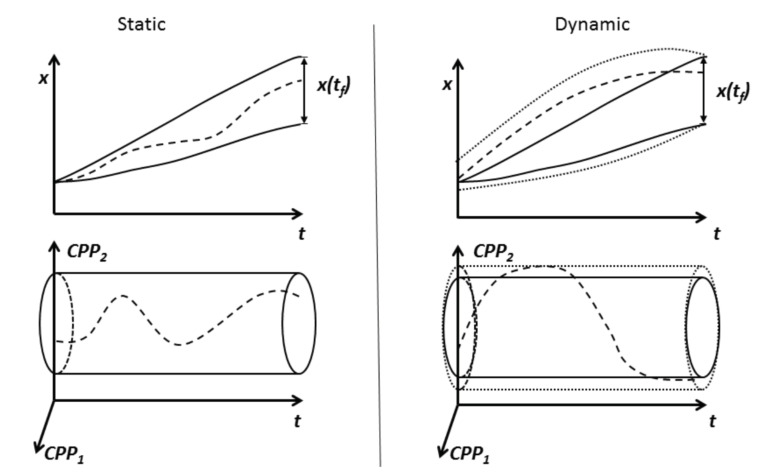
Illustration of the static and dynamic design space. Continuous lines: Static design space boundaries and the respective boundaries of the state (at the end of the process the boundaries of the state are assumed to be identical to the Critical Quality Attribute (CQA) acceptance limits). Dashed lines: Trajectory of the parameter and respective state trajectory for one process; i.e., *p* and x(t). Dotted Line: Dynamic design space boundaries; moving temporally outside means that the final CQA acceptance criterion can no longer be reached.

Equation (4) can be used instead of Equation (1) to describe the design space, and they allow us to assess the impact of temporal deviations on both the process dynamics and the final response surface. With this ansatz, the process state at each time instance is incorporated into the dynamic design space, which now embodies information on the overall evolution of the process. It is worth noting that a particular state might be reached from different initial conditions under the appropriate choice of temporal sequences of process parameters. The inclusion of the state into the design space representation might allow for larger variations in the CPPs and CMAs [50], as exemplified in Figure 2. Thus, to allow more variation in these variables it gives more flexibility to control the process, while still ensuring that the final CQAs fulfill the acceptance criteria and might even result in improved process operation strategies [57]. A number of dynamic design space approaches have already been proposed (see Table 1), yet training, as well as the mindset of people in the industry, seems to revolve around the development of static design space models, without realizing its limitations.

### 3.1. Suitable Representations of the Dynamic Process Design Space for Process Operation and Approval Filing

During operation as well as during optimization of the operation strategy, it must be ensured that the predicted CQAs are always within the defined CQA space (see Section 2.2). This can be achieved by (1) imposing constraints on the predicted CQAs to always remain within the defined CQA space; or (2) translating the CQA space into a corresponding multidimensional space of the CPPs and CMAs, subsequently checking whether the CPPs and CMAs are within the specified space. The second case is explored in more detail in the following section on the determination of the design space boundaries. In the first case, the process design space can be defined by:

(5) $$\begin{array}{ccc} \frac{dx}{dt} & = & g\left(x,\mathrm{CPPs}(t),\mathrm{CMAs}(t),p\right) \end{array}$$

(6) $$\begin{array}{ccc} x_{0} & = & x\left(t_{0}\right) \end{array}$$

(7) $$\begin{array}{ccc} \mathrm{CQAs}(t) & = & h\left(x(t),\mathrm{CPPs}(t),\mathrm{CMAs}(t),\omega \right) \end{array}$$

(8) $$\begin{array}{ccc} e(\mathrm{CQA},\beta ) & \leq & 0. \end{array}$$

Here e(·) is the approach describing the defined CQAs space, β is a set of parameters that characterize the limits on the acceptable criteria for the CQAs and $t_{0}$ is the initial process time. This set of equations could readily be implemented into advanced process control packages, and apart from explicitly accounting for the dynamics, it is similar to those proposed by [11,13,61].

### 3.2. Approaches to Dynamic Design Space Exploration

The process environment and operation are of dynamic nature, which is also reflected in the formulation “operating within the design space” that can be found in the ICH Q8 guidance. In light of this dynamic nature, approaches that explore the process dynamics and allow for the derivation of a dynamic model (that can be used for both, characterization of the design space and process control) seem warranted. While dynamics are well explored in the area of process control and while this area also describes approaches for the identification of the process transfer function (i.e., how the process outputs respond to changes in the inputs), the systematic exploration of multidimensional spaces appears to be inadequately covered in the process control literature.

Recently, an intensified Design of Experiments (iDoE) approach was proposed [63,64], which combines a statistical DoE with a system identification approach, namely step changes. In particular, two or more conditions (points) of a DoE are evaluated in one experiment, changing from one condition to the next after some predefined time, i.e., making a step-change potentially in more than one CPP. While the process response needs to be captured experimentally (using an adequate sampling strategy [64]), the dynamic changes need to be considered for the analysis of the response, e.g., by using a dynamic model. Besides the potential to capture process dynamics, the iDoE approach also has shown to require significantly fewer experiments for modeling the process (up to a factor of three) than classical DoE approaches [63,64]. In another approach, referred to as the Design of Dynamic Experiments (DoDE) [65], dynamic profiles are defined for a subset of factors, and the response is modeled by incorporating the integrated dynamic profiles into a response surface type model. It seems possible to use also the DoDE approach for the development of a dynamic model, which can then be used for advanced process control. Model-based (optimal) experimental design is yet another alternative that could help to investigate the dynamics and design space. Different strategies can be followed with the implementation of the model-based design, e.g., updating the model parameters, discriminating between competing model candidates, exploring the process region where the process optimum is assumed. This is in particular interesting in light of the aim of a higher degree of laboratory automation and the potential to operate the high-throughput equipment dynamically (e.g., changing the feed rates during the runs), see examples [66,67].

### 3.3. Determination of the Design Space Boundaries—Edge of Failure

In the case of the static design spaces Equation (1), the boundaries of the design space can be relatively easily assessed [68,69] though the impact of uncertainty should be accounted for to reduce the possibility that at the borders of the design space the CQA acceptance criteria are not met. For instance, a weighted normal distribution could be used for each process parameter to simulate the uncertainties, estimating the rate of defects or the proportion of the response variable outside the acceptance limits, without the need of a large number of simulations. The boundaries of the design space would then be chosen such that the overall rate of defects is smaller than a given threshold (e.g., 5%). Several approaches have been proposed to this end, e.g., Monte-Carlo simulations, Bayesian posterior predictive intervals, prediction, and tolerance intervals [7,70,71,72].

For dynamic design spaces, the determination of the boundaries requires the consideration of all feasible combinations of initial values ($x_{0}$), CPPs, and CMAs to translate the CQA space. Several authors [17,57,61] propose to perform grid evaluations, i.e., to draw samples for every time point from a grid of process parameters and assess the possibility of failure. This approach, being conceptually simple, suffers from two weaknesses. First, as the number of process parameters increases, the number of process conditions that have to be assessed becomes prohibitively large; this number scales up as a factorial. Second, this approach offers a static time-slot in a continuous process. A more robust approach would account for the dynamics as well; in other words, it is not important to define just the boundaries but also the rates with which trajectories approach them.

Advances in the mathematical and numerical analysis of ordinary differential equations can be employed for identifying the reachability of states given a set of initial conditions and a time window [73]. The idea is to combine a traditional Lyapunov stability analysis with rigorous numerical simulations and determine the space-time cylinder within which the solution profiles live. Note that the term stability is used here in the process control context of system stability, i.e., does the state return to a particular state upon excitation. Translating into our problem, this amounts to detecting CPP trajectories such that the CQA acceptance criteria at the end of the process (or of the process unit operation) are still met. In fact, as the properties of the system are determined by the functional form g, for simple expressions, it is actually possible to a priori determine reachability in closed-form and thus save computational time. A mathematical representation of the translated and explored combinations could maybe be obtained by using some of the approaches described in the section on alternative representations of multidimensional spaces.

The concept of reachability could also be employed for the identification of trajectories that are more at risk than others. In light of this, the integration of uncertainties into the concept of reachability could be interesting. Yet another potentially suitable approach is the combination of a confidence interval criterion (that can be computed for the model predictions) with a criterion that checks the distance of process points from the ones used during the development of the design space model [8,64], and associated risk according to the distance. For points lying far enough (high risk), retrospective experiments might be required to verify that the CQA meets the acceptance criterion. In terms of continuous improvement, every newly investigated process could be added to the design space, thereby extending the domain in which the process is understood and can be modeled.

### 3.4. Verification of the Design Space

Design space verification or validation means that it must be demonstrated that process operation within the design space is capable of manufacturing quality products at a commercial scale, implying that within the design space, scale-up effects are considered [7]. Two approaches for design space and model validation are proposed, i.e., an active and passive approach. In the active approach, the model is used to design a new experiment, and the obtained experimental response is compared to the predicted one, where a close match should be observed for the model to be valid. Here, the meaning of “close match” can vary from case to case as it might depend on several different factors, such as process to process variation, variation in the analytic quantification. The computation of a proper interval (e.g., prediction interval) should be performed as a reference to determine the “close match” of the new results to the predicted mean. In the passive approach, a set of experiments that have already been executed but that have not been used for model development are applied for checking the model predictions against the measured values. Both approaches can directly be adopted for the validation of dynamic design spaces and models; see, for instance, [74] for an evaluation of the validity of a dynamic model.

In the case that scale-down models (i.e., physical representation) are used to study the behavior of the system at a commercial scale, the potential changes in the dynamics, which are due to the scale-up, might have to be carefully considered. While this adds some complexity to the development and validation of the scale-down model, it might potentially increase the representativeness or help better to understand the limitations of scale-down “models”. Note that the task of showing that the scale-down “model” is representative of the commercial scale is not addressed here, nor are strategies for scalability of the design space discussed in more detail, but we merely advise to consider the process dynamics for design space verification. In terms of scaling effects, the reader is referred to detailed process unit-specific discussions on establishing representative scale-down or -up models [75,76,77,78,79,80] and FDA’s Questions and Answers on Design Space Verification [81].

## 4. Conclusions

The representation of the design space consists of two parts: (1) a model that links the CPPs and CMAs to the CQAs; and (2) a constraint that specifies whether the model-predicted CQAs are within the CQAs space meeting the acceptance criteria. It was shown that dynamic modeling approaches should be adopted for part 1, because (a) they offer more flexibility to operate; and (b) they allow integration of process characterization and control strategy development activities. The implications for the exploration and validation of the design spaces have been highlighted, and approaches for determining the design space boundaries have been discussed, underlining that there is a need for further research. From a regulatory perspective, the described approaches should be acceptable, if not more favorable, as they show an increased understanding of the process and especially its dynamics. In addition, it is explicitly stated in the ICH Q8 guideline [6] that: “A design space can be described in terms of ranges of material attributes and process parameters, or through more complex mathematical relationships. It is possible to describe a design space as a time-dependent function, or as a combination of variables such as components of a multivariate model.”

## Figures and Tables

**Table 1:** Publication reporting Dynamic Design Space approaches. The area of application, the dynamic modeling approach and the design space characterization and representation are detailed.

	Modeling Approach	Area of Application	Design Space Characterization and Representation
Burt et al. [58]	Combination of a material balance based set of ordinary differential equations with a statistical model describing the impurities	Chemical drug substance manufacturing process	DynoChem${}^{®}$ used for design space exploration, which seems to perform a grid-evaluation for design space characterization. Rectangular design space representation.
Fissore et al. [59]	Material and Energy balance based Ordinary Differential Equation	Freeze drying: Primary drying step	A grid evaluation technique for characterizing the design space and visual representation of the limits.
Adam et al. [60]	Material, Momentum and Energy balances solved using Discrete Element Method and Computational Fluid Dynamics	Pharmaceutical Blending Process	The model is exploited to create examples of contour plots for some CPPs but cannot be used for advanced process control.
García-Muñoz et al. [61]	(1) Mass balance in the packed bed column and scavenger particle level in form of Partial differential Equations (2) Mass balance of the Suzuki coupling reaction components in form of Ordinary Differential Equations	(1) Continuous Pd Removal in Packed Bed Columns (2) Suzuki Reaction	Grid-evaluation for design space characterization and uncertainty evaluation. Geometric representation of the Design Space.
Mortier et al. [17]	Material and Energy balance based Ordinary Differential Equation	Freeze drying: Primary drying step	Grid-evaluation for design space characterization and uncertainty evaluation.
Sun et al. [62]	Statistical modeling of a multi-unit operation process	Panax Notoginseng Saponins immediate release tablet	Multi-block partial least squares path model.
Vanbillemont et al. [44]	Material and energy balance based Ordinary Differential Equation	Freeze drying: Primary drying step	Supervisor process control for dynamic design space implementation under uncertainty.

## Abbreviations

The following abbreviations are used in this manuscript:

PAT	Process Analytic Technology
FDA	U.S. Food and Drug Administration
ICH	International Conference on Harmonization
QbD	Quality by Design
QbC	Quality by Control
DoE	Design of Experiments
iDoE	intensified Design of Experiments
DoDE	Design of Dynamic Experiments
QTPP	Quality Target Product Profile
CQA	Critical Quality Attributes
CPP	Critical Process Parameters
CMA	Critical Material Attributes
ANOVA	Analysis of Variance
MPC	Model Predictive Control
ODEs	Ordinary Differential Equations
SA	Sensitivity Analysis
PCA	Principal Component Analysis
PLS	Partial Least Square
